# Association of Serum Symmetric Dimethylarginine Concentrations and Inflammation in Cats

**DOI:** 10.1111/jvim.70030

**Published:** 2025-02-26

**Authors:** Giulia Cattaneo, Edward J. Kingsbury, Katie E. McCallum, Tim L. Williams

**Affiliations:** ^1^ Department of Veterinary Medicine, The Queen's Veterinary School Hospital (QVSH) University of Cambridge Cambridge UK

**Keywords:** feline, SAA, SDMA, systemic inflammation

## Abstract

**Background:**

Serum symmetric dimethylarginine (SDMA) concentrations are higher in some hyperthyroid cats with normal renal function, presumably due to increased protein catabolism.

**Objectives:**

To investigate if SDMA is higher in cats with inflammation (defined as elevated serum amyloid A [SAA]).

**Animals:**

Twenty‐eight cats: 12 with elevated SAA concentrations (> 3.9 μg/mL) and 16 with normal SAA.

**Methods:**

Retrospective case control study. Cats presenting to a referral institution between 2016 and 2022 with a documented SAA were identified. Individuals with renal and extrarenal factors known to affect SDMA were excluded. SDMA was measured from stored serum samples. Comparisons were made using the Mann–Whitney *U* test, and correlations assessed using Spearman's correlation coefficient. Data are presented as median [minimum–maximum].

**Results:**

SDMA was not significantly different between cats with elevated SAA and normal SAA (11 [5–17] μg/dL vs. 13 [9–21] μg/dL, respectively; *p* = 0.28). There was no correlation between SDMA and SAA (*r*_s_ = −0.105; *p* = 0.594) or serum TT4 concentrations (*r*_s_ = −0.023; *p* = 0.906). No difference in age or USG was present between elevated SAA and normal SAA groups (*p* = 0.908 and *p* = 0.165, respectively). Serum urea and creatinine concentrations were both significantly lower in cats with elevated SAA compared to those with normal SAA (6.3 [3.6–8.8] mmol/L vs. 8.4 [6.2–10.5] mmol/L; *p* = 0.008, and 96 [62–129] μmol/L vs. 118 [90–147] μmol/L; *p* = 0.008, respectively).

**Conclusions and Clinical Importance:**

SDMA might be a more representative biomarker of GFR during inflammatory states, provided other confounding factors that affect SDMA are eliminated.

## 1 Introduction

Symmetric dimethylarginine (SDMA) is a by‐product of intranuclear arginine methylation in all nucleated cells, specifically by Type II protein arginine methyltransferase (PRMT) enzymes [1], and is released during proteolysis. It is primarily excreted by renal filtration [2] and serum SDMA concentrations are less influenced by changes in muscle mass than are serum creatinine concentrations [3]. In cats with chronic kidney disease (CKD), serum SDMA is inversely correlated to glomerular filtration rate (GFR) [4] and might be a more sensitive marker of CKD than serum creatinine concentrations [2]. However, one study was unable to demonstrate the superiority of serum SDMA over serum creatinine concentrations in cats with CKD [5]. SDMA is a biomarker of acute kidney injury (AKI), although serum SDMA alone is unable to distinguish between AKI and CKD [6]. In addition, prerenal azotemia and alterations in volume status such as dehydration also increase SDMA [7].

Recent literature suggests that extrarenal factors or comorbidities might influence serum SDMA; for example, a proportion of dogs and cats with lymphoma have higher serum SDMA (in absence of higher creatinine concentrations) compared to controls, possibly due to overexpression of PRMT enzymes in these neoplasms [8]. This is also reported in leukemia, lymphoma, and other solid tumors in humans [9, 10, 11]. Furthermore, cats with diabetes mellitus have significantly lower serum SDMA when compared to cats with renal disease, cardiac disease, and healthy controls, most likely secondary to hyperfiltration and osmotic diuresis [12]. Finally, hyperthyroidism has variable effects on serum SDMA, with higher serum SDMA observed in cats after radioiodine treatment for hyperthyroidism, likely reflecting the treatment‐associated reduction in GFR [13, 14]. In contrast, other studies describe decreases in serum SDMA after radioiodine treatment while also highlighting a discordance with serum creatinine concentrations [15, 16], possibly reflecting shifts in protein catabolism and the effects of hyperthyroidism on muscle mass, respectively. Furthermore, the relationship between serum SDMA and serum creatinine concentrations in hyperthyroid cats treated by bilateral thyroidectomy, although linear, is steeper (i.e., SDMA is relatively higher) in those that remain hyperthyroid after treatment compared with those that become euthyroid or hypothyroid. This could reflect increased SDMA production secondary to hypercatabolism and reduced creatinine production secondary to reduced muscle mass in hyperthyroid cats [17]. Hyperthyroidism should therefore be considered a confounding factor when interpreting serum SDMA in cats.

Similar to hyperthyroidism, inflammation is another process associated with increased protein catabolism; hence, it has been postulated that this might also increase serum SDMA [18]. In human intensive care unit (ICU) patients, serum SDMA is positively correlated with markers of systemic inflammation, including white blood cell concentrations, and serum concentrations of C‐reactive protein (CRP), procalcitonin, and tumor necrosis factor [18]. Only one small study has investigated serum SDMA in dogs with systemic inflammation [19], and this study does not identify significant differences in serum SDMA between dogs in ICU and healthy control dogs; however, 4 of 12 dogs (2 of which were also non‐azotemic) with severe illness (based on an acute patient physiologic and laboratory evaluation [APPLE] score > 30) had serum SDMA above the laboratory reference interval, perhaps suggesting that severe systemic inflammation might be associated with serum SDMA, as in human patients. The effects of inflammation on serum SDMA in cats have not yet been evaluated. In the present study, we investigated serum SDMA in cats in inflammatory states, as defined by elevated serum amyloid A (SAA) concentrations; we hypothesized that cats with elevated SAA would have higher serum SDMA than cats with normal SAA.

## 2 Materials and Methods

### 2.1 Case Selection

Cases that had attended a university teaching hospital between 2016 and 2022 with recorded SAA concentrations were eligible for inclusion. Other quantitative data including age, biochemical parameters (serum concentrations of total thyroxine [TT4], urea, creatinine, glucose), urinalysis findings (urine specific gravity [USG] and dipstick findings) and qualitative data (signalment, pertinent history, final diagnosis) were also collected.

To rule out the effects of renal or prerenal factors that have been reported to affect SDMA, cats with azotemia, defined as elevated serum concentrations of urea (> 10.7 mmol/L; upper limit of laboratory‐specific reference interval) and/or creatinine (≥ 140 μmol/L, corresponding to serum creatinine concentrations expected in AKI/CKD IRIS Stage 2 or above), were excluded [2, 4, 7, 20, 21]. In addition, only cats with adequate urine concentrating ability (USG ≥ 1.035) within 7 days of SAA measurement were eligible for inclusion [20, 21]. Cases with extrarenal factors or comorbidities known to influence serum SDMA, such as overt or suspected hyperthyroidism (TT4 ≥ 35 nmol/L), neoplasia, and diabetes mellitus were excluded. Cats administered corticosteroids at the time of blood sampling or within 7 days before sampling were omitted on the basis that corticosteroid therapy could affect protein catabolism and renal values [22, 23, 24]. Where possible, additional data pertaining to body condition score (BCS), hospitalization, use of intravenous fluid therapy (IVFT) and nutritional status in each cat were collected.

Cases were then divided further into individuals with elevated SAA, defined as SAA concentrations > 3.9 μg/mL [25], or with normal SAA that had SAA concentrations < 3.9 μg/mL.

### 2.2 SAA Analysis

Measurement of SAA concentrations was performed in‐house on serum within 2 h of sample collection. A previously validated human turbidimetric immunoassay (TIA) for SAA was used (LZ‐SAA, Eiken Chemical Co., Tokyo, Japan) [25]. SAA concentrations below the limit of blank of the assay (< 0.3 μg/mL) were defined as 0.15 μg/mL for the purposes of statistical analysis. For cats that had multiple serum SAA concentrations determined, the highest concentration was selected, providing the previously described selection criteria were also adhered to.

### 2.3 SDMA Analysis

Measurement of serum SDMA was performed using archived serum samples. Blood samples were collected from cats and serum was harvested within 2 h of sampling. Residual serum was frozen within 8 h of biochemical analysis and stored on‐site at a temperature of −80°C. Serum SDMA concentrations were measured at a reference laboratory using a previously described immunoassay method (IDEXX Laboratories, Wetherby, UK) [26].

### 2.4 Statistical Analysis

Data are presented as median [minimum–maximum], unless otherwise specified. Commercially available software (SPSS v27 and GraphPad Prism) was used to carry out the statistical analysis. Comparisons between groups were made using the Mann–Whitney *U* test, and correlations were assessed using Spearman's correlation coefficient. Statistical significance was defined as *p* < 0.05.

## 3 Results

In the initial data search, 1455 individual SAA concentrations from 890 cats were obtained. Of these cats, 443 were excluded due to lack of urinalysis, 220 with USG < 1.035, and 51 due to elevations in urea and/or creatinine concentrations. Seventy‐eight cases were further excluded due to incomplete datasets (including lack of biochemistry results, stored serum samples, or unclear data available) and the remainder due to the presence of extrarenal factors including neoplasia (*n* = 25), diabetes mellitus (*n* = 21), and elevated total T4 (*n* = 19) which affect serum SDMA. A further five cases were later excluded due to documented corticosteroid use at the time of, or immediately before, SAA assessment. Twenty‐eight cats were included in the study: 12 cats with elevated SAA (serum concentrations of 152.6 [10.9–263.2] μg/mL) and 16 cats with normal SAA (serum concentrations of < 0.3 [< 0.3–2.2] μg/mL).

The cats were 6.7 [0.5–13.4] years of age, and the predominant breed was Domestic Short Hair (*n* = 18) followed by Bengal (*n* = 4), Maine Coon (*n* = 2), Persian (*n* = 2), Oriental Shorthair (*n* = 1), and British Shorthair (*n* = 1). The most common final diagnoses (Table 1) were gastrointestinal disease (including pancreatitis and cholangiohepatitis; *n* = 10) and feline infectious peritonitis (FIP; *n* = 5). Urogenital disease (*n* = 3), neurological disease (*n* = 3), pyrexia of unknown origin (PUO; *n* = 2), and other final diagnoses (*n* = 5; hypertrophic cardiomyopathy, mycobacterial disease [*n* = 2], idiopathic hypercalcemia, pemphigus foliaceous) were also documented.

**TABLE 1:** The final diagnoses in cats with elevated and normal SAA (as defined by serum amyloid A concentrations > 3.9 or < 3.9 μg/mL, respectively) that were included in the study.

Final diagnoses	Normal SAA	Elevated SAA	Total
Gastrointestinal disease (including pancreatitis and cholangiohepatitis)	7	3	10
FIP	0	5	5
Urogenital disease	3	0	3
Neurological disease	2	1	3
Pyrexia of unknown origin (PUO)	1	1	2
Other	3	2	5
Total	16	12	28

**FIGURE 1:**
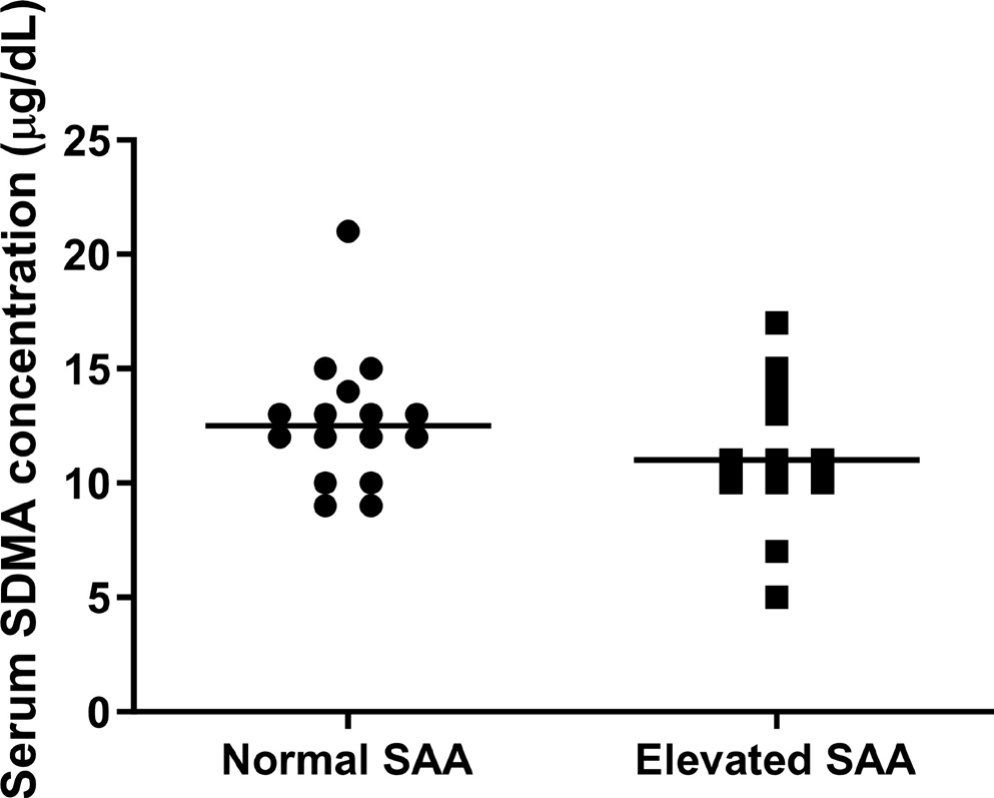
Scatter plot of serum SDMA measurements in cats with normal SAA versus elevated SAA. Comparisons between groups were made using the Mann–Whitney *U* test. The line represents the median concentration in each group. Serum SDMA was not significantly different between cats with elevated and normal SAA (*p* = 0.28).

**FIGURE 2:**
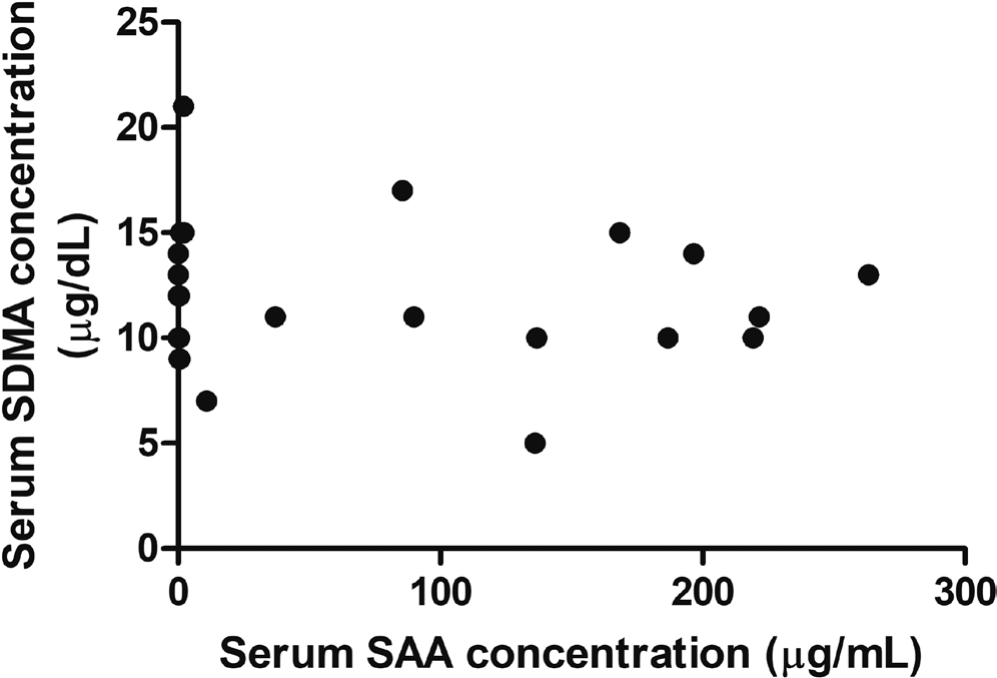
Scatter plot of serum SAA concentrations vs. serum SDMA concentrations. Correlations were assessed using Spearman's correlation coefficient. No significant correlation was observed between these two groups (*r*_s_ = −0.105; *p* = 0.594).

**FIGURE 3:**
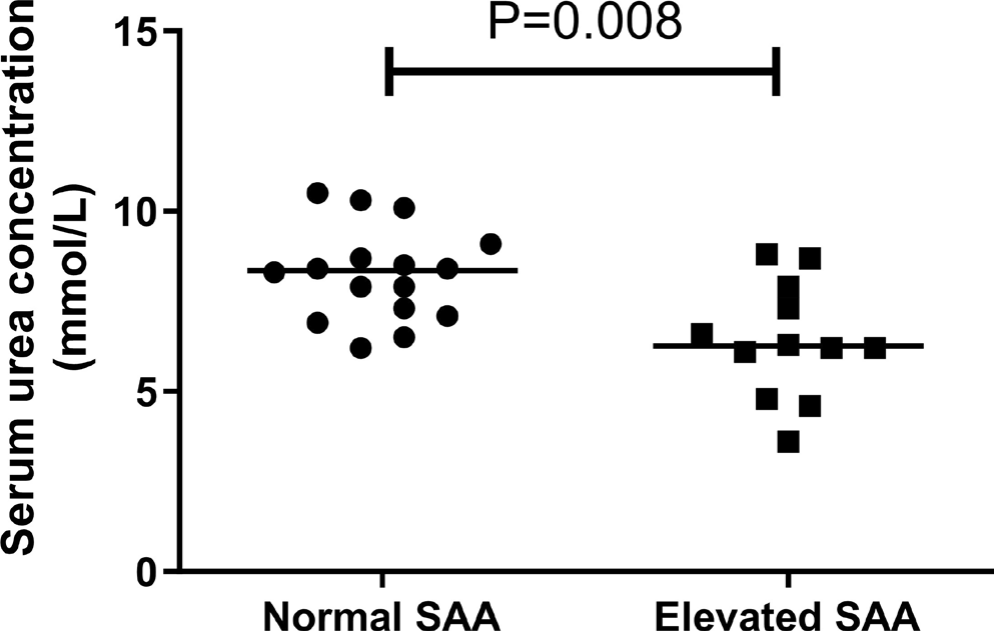
Scatter plot of serum urea concentrations in cats with normal SAA versus elevated SAA. Comparisons between groups were made using the Mann–Whitney *U* test. The line represents the median concentration in each group. Serum urea concentrations were significantly lower in cats with elevated SAA when compared to cats with normal SAA (*p* = 0.008).

**FIGURE 4:**
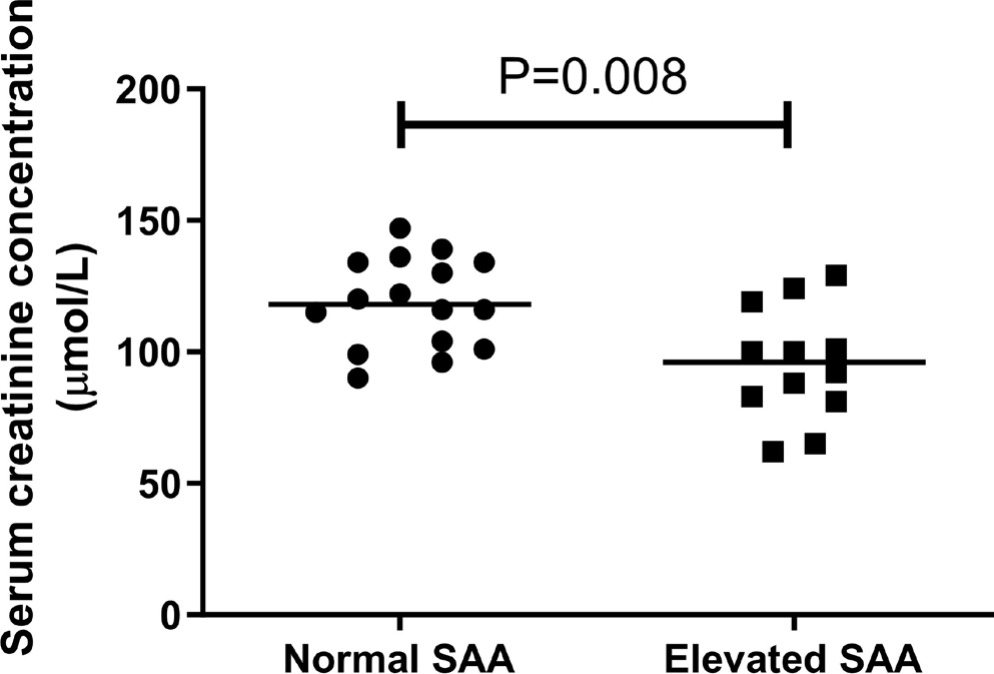
Scatter plot of serum creatinine concentrations in cats with normal SAA versus elevated SAA. Comparisons between groups were made using the Mann–Whitney *U* test. The line represents the median concentration in each group. Serum creatinine concentrations were significantly lower in cats with elevated SAA when compared to cats with normal SAA (*p* = 0.008).

Serum SDMA was not significantly different between cats with and without elevated SAA (11 [5–17] μg/dL vs. 13 [9–21] μg/dL, respectively; *p* = 0.28; Figure 1). No correlation was noted between serum SDMA and SAA concentrations (*r*_s_ = −0.105, *n* = 28; *p* = 0.594 Figure 2) or serum TT4 concentrations (*r*_s_ = −0.023, *n* = 28; *p* = 0.906). Comparisons of selected variables between the elevated SAA and normal SAA groups are summarized in Table 2, with no difference in age or USG present between the two groups (*p* = 0.908 and *p* = 0.165, respectively). However, serum urea and creatinine concentrations were both significantly lower in cats with elevated SAA compared to those with normal SAA (*p* = 0.008; Table 2; Figures 3 and 4).

**TABLE 2:** Selected clinicopathological variables in the elevated SAA and normal SAA groups. Comparisons between groups were made using the Mann–Whitney *U* test.

	Normal SAA		Elevated SAA		*p*
	Median	Min–max	Median	Min–max	
Urine specific gravity	1.042	1.035–1.050	1.045	1.035–1.053	0.165
Age (years)	6.1	0.5–13.4	6.3	0.7–11.5	0.908
Urea (mmol/L)	8.4	6.2–10.5	6.3	3.6–8.8	0.008
Creatinine (μmol/L)	118	90–147	96	62–129	0.008

Data pertaining to nutritional status, hospitalization, BCS, and use of IVFT are shown in Table S1. Based on a review of the clinical records from our hospital and the referring veterinarian (where available), the majority of cases were considered unlikely to have received IVFT before sampling for SAA/SDMA.

## 4 Discussion

The aim of this study was to investigate whether the presence of inflammation is a confounding factor for the interpretation of serum SDMA in cats, since one study in dogs reports higher SDMA in a small number of non‐azotemic dogs with severe illness [19]. Inflammation has also been investigated as a confounding factor for the interpretation of other biomarkers; for example, the cardiac biomarker NT‐proBNP is associated with circulating IL‐6 concentrations independent of other factors known to affect NT‐proBNP in humans [27]. This study of cats investigating the relationship between serum SDMA and inflammatory states did not find any association between inflammation (defined as elevated serum SAA concentrations) and serum SDMA. However, this study was likely statistically underpowered to detect significant differences between the groups. Based on the data in the present study (standard deviation of serum SDMA concentrations in all cats being 3.1 μg/dL), 38 cats per group are needed to have 80% statistical power to detect a 2 μg/dL higher mean SDMA in the elevated SAA versus the normal SAA group (and corresponding to a SDMA above the reference interval) at the 5% significance level, and so larger studies are required to confirm our findings. Serum SDMA remains unchanged during inflammation in humans [28] which supports our findings; however, it conflicts with other data in humans [18] which contends that inflammation (defined by CRP and other inflammatory biomarker assays, rather than SAA) is another factor associated with serum SDMA, independent of effects on renal function. There is also evidence to implicate SDMA itself in the pathogenesis of inflammation since in vitro studies document a reduction of endothelial nitric oxide (NO) synthesis by SDMA during inflammatory states, likely at the level of nitric oxide synthase (NOS) through competitive inhibition with l‐arginine [29]. Other human in vitro studies describe a role of SDMA in inducing vascular damage through increased reactive oxygen species (ROS) production by monocytes [30] and involvement in the inflammatory process of CKD by activating NF‐κB, subsequently resulting in enhanced expression of IL‐6 and TNF‐α [31]. The lack of correlation between serum SDMA and SAA concentrations in the present study would not support the existence of this relationship in cats.

Serum urea and creatinine concentrations were significantly lower in cats with elevated SAA when compared to those with normal SAA in the present study. We hypothesize that the lower serum urea and creatinine concentrations reflect a reduction in dietary protein intake [32, 33, 34] and increased muscle catabolism or lean muscle loss [35], respectively, secondary to inflammation. It should also be noted that the effect of dietary protein intake and muscle catabolism on renal variables in cats is complex, with multiple, potentially opposing influences. In fact, the feeding of a high protein diet results in higher serum urea concentrations but lower serum creatinine concentrations [33], the latter presumably secondary to an increase in GFR associated with protein intake. Hence, based on the findings of the aforementioned study, it could instead be postulated that a low protein intake (hyporexia, anorexia) in diseased cats might decrease GFR and therefore lead to higher (rather than lower) serum creatinine concentrations. Conversely, the acute phase response is associated with increased proteolysis and muscle catabolism [36], which could increase urea production (and hence serum urea concentrations) while reducing lean muscle mass and serum creatinine concentrations. Therefore, assessment of the direct relationship between serum urea/creatinine concentrations and nutritional status/BCS of cats is likely to be challenging.

Renal function could also be directly influenced by inflammation per se, since a negative correlation between the proinflammatory cytokine IL‐17α and estimated GFR (eGFR) is found in healthy human volunteers in one study [37]. However, evaluation of the association between inflammation and GFR is likely to be confounded by concurrent AKI (azotemic or non‐azotemic) [20] which is a common sequela of systemic inflammatory response syndrome (SIRS) and sepsis, often as a consequence of critical illness‐related corticosteroid insufficiency and hypotension in these inflammatory states [38, 39, 40, 41]. These factors would impair renal blood flow and decrease GFR and hence render the detection of increased SDMA production secondary to inflammation based on changes in serum SDMA alone challenging. In severe sepsis, SDMA is related to the concentrations of inflammatory markers CRP and IL‐6; however, this is not independent of serum creatinine concentrations and so the relationship between SDMA and CRP likely reflects the presence of renal failure in septic patients [42], rather than a direct relationship between systemic inflammation and SDMA. Furthermore, any increase in SDMA secondary to increased proteolysis in inflammatory states could be opposed by an increase in GFR (if this occurs) leading to enhanced excretion of SDMA; the production of excess endogenous glucocorticoids due to upregulation of the hypothalamic–pituitary–adrenal (HPA) axis or their reduced metabolism during critical illness [36, 43] could have a part to play since glucocorticoids can also increase GFR [44]. However, the negative association between IL‐17α and eGFR [37], alongside the expected reduction in GFR associated with SIRS and sepsis, would suggest that inflammation should be associated with higher serum urea and creatinine concentrations. This contradicts the findings of the present study; hence, we would contend that the lower serum urea and creatinine concentrations observed in cats with elevated SAA reflect changes in dietary protein intake and body muscle mass in cats with systemic illness rather than an increase in GFR during inflammatory states. Since serum SDMA might be less influenced by inflammation than serum urea/creatinine concentrations, based on our initial data, the authors propose that serum SDMA might be a more reliable, indirect marker of GFR during inflammatory states, providing known renal and extrarenal factors that affect serum SDMA are excluded.

There were several limitations in the present study, one of which included the small sample size. This would increase the likelihood of statistical Type 2 error, and post hoc power calculations suggest that our study is statistically underpowered to detect a 2 μg/dL higher mean SDMA in the elevated SAA group versus normal SAA group should such a difference exist. Despite excluding a large proportion of the study sample that ultimately resulted in the small sample size, the stringent selection criteria were necessary in order to exclude confounding factors that are known to influence serum SDMA concentrations [2, 4, 6, 7, 8, 9, 10, 11, 12, 13, 14, 15, 16, 17, 20, 21] and enable meaningful comparisons. Given that many SIRS/sepsis cases present with concurrent AKI [20, 40] or prerenal azotemia [7], and cases with azotemia were excluded from the present study, it is also possible that the most severe cases of inflammation, in which SDMA production might be altered, were excluded. Given that the degree of systemic involvement of inflammation cannot be determined based on serum SAA concentrations alone, this could have biased our dataset toward cats with less severe inflammation, although distinguishing changes in serum SDMA due to changes in GFR versus changes in protein metabolism in such cats would be difficult for the reasons previously mentioned. Furthermore, in the present study, inflammation was defined based on detection of SAA concentrations above the laboratory reference interval, although it is important to note that elevated versus normal SAA concentrations are not synonymous with the presence of absence of inflammation, respectively. Some cats with inflammation might have had SAA concentrations within the reference interval (and vice versa), which might have confounded our results. The sensitivity and specificity of an elevated SAA concentration for inflammation will be dependent on the cut‐off value used, and it is possible that the use of a higher cut‐off value for inflammation might increase the specificity (whilst likely decreasing sensitivity) for detection of inflammation. However, given that there is inherent inter‐assay and inter‐laboratory variation in SAA concentrations, validation of an alternative cut‐off value would need to be undertaken in each laboratory and require assessment of other biomarkers for inflammation, such as serum concentrations of other acute phase proteins (e.g., alpha‐1‐acid glycoprotein) and/or consideration of clinical information, which was beyond the scope of the current study. This was also a retrospective study which possesses the inherent drawback of incomplete datasets and non‐standardization of variables pertaining to the individual, sampling, and case management; therefore, prospective studies with the recruitment of increased sample sizes and/or improved standardization would allow for the collection of more complete datasets, for example, with urine sampling performed in all cases. Direct measurement of GFR [45] was not available in the study which would have permitted us to exclude an increase in GFR as a cause of the observed reductions in serum urea and creatinine concentrations in animals with elevated SAA. Furthermore, the cats with normal SAA were not healthy individuals and so their concurrent disease processes might have impacted renal function (e.g., through induction of non‐azotemic AKI which could not be excluded fully based on our exclusion criteria) [20]. The use of IVFT and volume status [7] in hospitalized cats could have influenced urea, creatinine, and SDMA measurements; however, this data could not be reliably collected or correlated with the timing of blood and urine sampling given the retrospective nature of the study. In addition, serum concentrations of SAA might vary throughout the time course of an inflammatory process, which might have influenced the categorization of cats according to presence/absence of inflammatory disease in the present study and confounded analysis of the differences in SDMA between the groups. Finally, the effects of chronic disease on metabolism, which could affect serum urea and creatinine concentrations, might be relevant; factors such as the duration of the inflammatory state, muscle condition scoring, and nutritional status of the cats in future prospective studies should be evaluated as potential confounding factors.

In conclusion, this study did not find any association between elevated SAA and serum SDMA concentrations in cats, although cats with elevated SAA did have lower serum urea and creatinine concentrations than cats with normal SAA. The authors propose that serum SDMA concentrations might be a more reliable, indirect marker of GFR during inflammatory states in cats, provided other renal or extrarenal factors known to affect SDMA are eliminated.

## Disclosure

Authors declare no off‐label use of antimicrobials.

## Ethics Statement

Approved by the University of Cambridge Ethics and Welfare Committee, and The European College of Veterinary Clinical Pathology (ECVCP). Authors declare human ethics approval was not needed.

## Conflicts of Interest

The authors declare no conflicts of interest.

## Supporting information


**Table S1.** Table summarizing breed, sex, nutritional status, body condition score, hospitalization, intravenous fluid therapy (IVFT) and final diagnosis in all cases.
