# Supplementary material for: Association of Serum Symmetric Dimethylarginine Concentrations and Inflammation in Cats
Source: J Vet Intern Med. 2025 Feb 26;39(2):e70030. doi: 10.1111/jvim.70030 (PMC11863204; doi:10.1111/jvim.70030)
Supplement: Supplementary file 1 — Table S1. Table summarizing breed, sex, nutritional status, body condition score, hospitalization, intravenous fluid therapy (IVFT) and final diagnosis in all cases. [file JVIM-39-e70030-s001.docx]

**Supplementary Table 1:** Table summarizing breed, sex, nutritional status, body condition score, hospitalization, intravenous fluid therapy (IVFT) and final diagnosis in all cases.

Abbreviations: DSH, Domestic Shorthaired; MN, male neutered; ME, male entire; FN, female neutered; FE, female entire.

Where no data was available, this has been indicated with ‘-‘. Exact timings of blood sampling with respect to intravenous fluid therapy (IVFT) could not be determined. Where it is unclear, cases deemed to be ‘unlikely’ to have received IVFT before blood sampling are stated as such given that hospitalization of the cat was likely preceded by blood sampling in most cases.

|  | **Breed** | **Sex** | **Nutritional status/presenting clinical signs** | **Body condition score (out of 9)** | **Hospitalization** | **Intravenous fluid therapy (IVFT) before blood sampling** | **Final diagnosis** |
| --- | --- | --- | --- | --- | --- | --- | --- |
| **Cats with elevated SAA** |  |  |  |  |  |  |  |
| 1 | DSH | MN | Chronic anorexia, lethargy | - | Yes | Unlikely | Suspected FIP |
| 2 | Maine Coon | FN | - | - | Yes | - | Pemphigus foliaceous |
| 3 | DSH | MN | Chronic inappetence, hyporexia, weight loss | 2 | No | - | Suspected FIP |
| 4 | Bengal X | MN | Chronic inappetence and lethargy | - | No | No | Suspected FIP |
| 5 | DSH | MN | Chronic inappetence and weight loss | 4 | No | No | Suspected FIP |
| 6 | British Shorthaired | FN | Weight loss | 3 | - | - | Pyogranulomatous osteitis |
| 7 | DSH | MN | Normal appetite | - | - | - | Cervical myelopathy |
| 8 | DSH | FN | Acute onset anorexia, lethargy | 4 | Yes | Unlikely | PUO |
| 9 | DSH | MN | Normal appetite, 36h vomiting | 5 | Yes | Unlikely | Acute enteropathy |
| 10 | DSH | MN | Missing for 1 year, weight loss noted | 3.5 | No | Yes | Collapse, vomiting, diarrhoea of unknown cause |
| 11 | Oriental Shorthaired | MN | Chronic hyporexia, lethargy | 3 | Yes | Unlikely | Dry FIP |
| 12 | DSH | FN | Acute onset hyporexia, lethargy | 4 | Yes | Unlikely | Acute pancreatitis |
| **Cats with normal SAA** |  |  |  |  |  |  |  |
| 1 | Persian | MN | - | - | - | - | Urolithiasis |
| 2 | DSH | FN | Normal appetite, chronic large intestinal diarrhoea and vomiting | 5 | No | No | Chronic enteropathy |
| 3 | Persian | MN | Normal appetite, intermittent dysuria and pollakiuria | 4 | No | No | Idiopathic hypercalcaemia |
| 4 | DSH | FN | Waxing and waning chronic intermittent hyporexia, pyrexia | 4 | Yes | Unlikely | Pyrexia of unknown origin (PUO) |
| 5 | Maine Coon | MN | Chronic weight loss and progressive inappetence | 3 | No | No | Possible previous bacterial cholangitis |
| 6 | Bengal | FN | No change to appetite, seizure-like events | 5 | No | No | Idiopathic seizures |
| 7 | DSH | MN | Acute onset hyporexia-anorexia, gagging, coughing, retching and lethargy | 4 | Yes | Unlikely | Intestinal parasitism |
| 8 | Bengal | MN | Acute onset hyporexia, diarrhoea | - | Yes | Unlikely | Resolving gastroenteritis and pancreatitis |
| 9 | DSH | ME | Normal appetite, faecal and urinary incontinence, paraparesis | - | No | No | Vertebral deformity leading to faecal/urinary incontinence and paraparesis |
| 10 | DSH | MN | Normal appetite, recurrent urethral obstruction | 8 | Yes | Unlikely | Feline idiopathic cystitis (FIC)/feline lower urinary tract disease (FLUTD) |
| 11 | DSH | FN | - | 5 | Yes | No | Hypertrophic cardiomyopathy (HCM) |
| 12 | DSH | MN | Normal appetite, chronic lethargy, incidental submandibular nodules | 5 | - | No | Mycobacteriosis |
| 13 | DSH | FN | Acute onset anorexia and vomiting | 9 | Yes | Yes | Pancreatitis |
| 14 | Bengal | FE | Normal appetite, chronic intermittent vomiting and acute large intestinal diarrhoea | - | Yes | Unlikely | Gastric foreign body |
| 15 | DSH | FN | Normal appetite, chronic intermittent vomiting and diarrhoea | 4 | - | Unlikely | Cholecystolith, chronic enteropathy |
| 16 | DSH | FN | No changes to appetite, acute onset difficulty urinating and loss of tail tone | 3.5 | Yes | Yes | Uroabdomen |
